# Ethical research in global health emergencies: making the case for a broader understanding of ‘research ethics’

**DOI:** 10.1093/inthealth/ihaa053

**Published:** 2020-11-09

**Authors:** Katharine S Wright

**Affiliations:** Nuffield Council on Bioethics, 28 Bedford Square, London WC1B 3JS, UK

**Keywords:** emergencies, ethics, global health, research

## Abstract

The ethical challenges of global health research become particularly acute in emergency contexts, and are exacerbated by historic inequities and imbalances in power and influence. Drawing on the findings of an international working group established by the Nuffield Council on Bioethics, this article argues for the need to take a broader approach to ‘research ethics’ as traditionally understood, to include the role of ‘duty-bearers’ such as funders, governments, research institutions and journals. An ‘ethical compass’ of three core values (equal respect, fairness and helping reduce suffering) supports ethical reflection at the level of policy, as well as on the ground.

## Introduction

The ethical challenges of global health research become particularly acute in the context of global health emergencies–from infectious disease outbreaks that require cross-border collaboration, to humanitarian crises caused by natural or human-made disasters. Such emergencies involve disruption, uncertainty and distress, as well as great health needs, increasing the difficulties involved in maintaining respectful and non-exploitative relationships between researchers and participants. Time pressures to act urgently are likely to be at variance with standard research timeframes. These challenges of conducting research ethically during an emergency are exacerbated further by the involvement of many different organisations, with potentially conflicting goals, and scope for tension over control and legitimacy.

**Figure 1.**
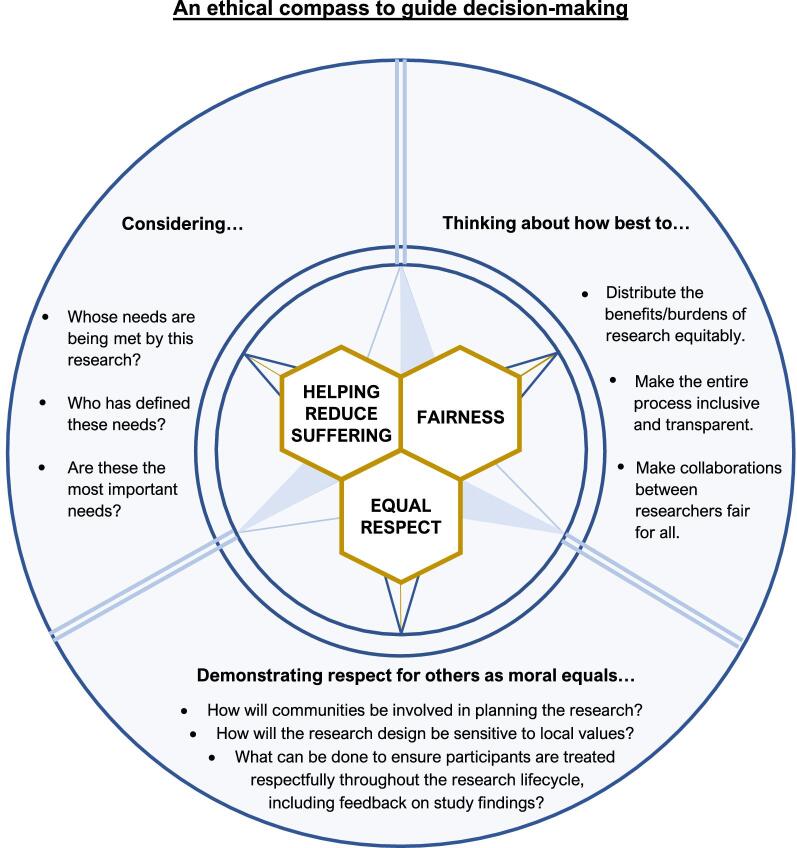
Nuffield Council on Bioethics' ethical compass to guide decision-making.

In January 2020, the UK-based Nuffield Council on Bioethics published a report by an international working group exploring these ethical challenges and making recommendations to policymakers.^1^ This commentary explores one of the report's key findings: that there needs to be a broader approach to what has traditionally been considered to constitute ‘research ethics’. This field of ethics is often narrowly construed as relating only, or primarily, to independent ethical review systems, with a particular focus on the scrutiny of consent processes. However, ethical considerations cannot be confined to one-off processes of review, and nor can they be considered in a vacuum. Rather, they arise throughout the lifecycle of research, from setting funding priorities to sharing and implementing findings. They are highly dependent on context; and are the responsibility of many actors in the research sector. These wider ethical considerations emerge with particular acuteness during emergencies–but are also relevant in research more broadly.

## Materials and methods

In a two-year project starting in early 2018, the multidisciplinary working group drew on the rich experiences of researchers, practitioners, research participants and policymakers from over 30 countries across six continents.^2^ The group held an open call for evidence, organised roundtable discussions in the UK, Philippines, Lebanon, Senegal and China, participated in international conferences and used a snowballing approach to carry out rapid literature reviews of community agency and engagement in four contrasting emergencies (Indian Ocean tsunami, triple disaster in Fukushima, Hurricane Katrina and conflict in Syria). The working group's report was reviewed by 13 international experts prior to publication.

## Results and discussion

Emergencies, by their nature, are challenging environments in which to conduct health-related research, because of the disruption, uncertainty, time pressure and distress described above. Crucially, such research also often takes place in the context of deep historical inequities and ongoing imbalances of power – indeed, neglect of the needs of marginalised groups may itself be the root cause of some emergencies.^3^ People who are most vulnerable, through poverty, lack of access to healthcare or education or political marginalisation, are disproportionately affected by emergencies.^4^ Yet these are the very groups who are least likely to have their voices heard with respect to the conduct of research carried out purportedly for their benefit.

In considering whether a proposed study can be conducted ethically in such circumstances, it is necessary, but not sufficient, to find context-sensitive ways of addressing ‘traditional’ research ethics questions, such as appropriate study design, independent scrutiny and meaningful consent processes. Crucially, ethics also needs to take account of the political and structural factors that shape people's lives and their interactions with the research process – whether as participants, healthcare workers or researchers. Ethical considerations thus additionally include:

- finding ways to ensure that the voices of those most affected can be meaningfully included in deciding what research takes place, where and how;
- achieving greater fairness in collaborations between researchers and institutions in different countries; and
- identifying ways in which frontline research workers can be better supported in addressing the ethical dilemmas they face.

Issues of study design, independent review and participant recruitment focus primarily on the duties of researchers and ethics committees, and on the interactions between researchers and participants. The broader ethical concerns outlined above, by contrast, bring in many other ‘duty-bearers’, ascribing duties on the basis of those who have the greatest ability to act, such as funders and governments, or those with responsibilities arising out of special relationships, such as employers/employees.

In order to guide ethical conduct at the level of policy, as well as on the ground, the report presents an ‘ethical compass’ of three core values: ‘**equal respect**’ (respect for others as moral equals), **fairness** and **helping reduce suffering** (Figure 1). The extent to which researchers on the ground can act in accordance with these values will often be affected by factors out of their control, arising out of institutional, funding or publication policies. The report makes the case for research funders, governments, research institutions, journals and others to recognise their role as duty-bearers, and take proactive steps to ensure that the research they fund, support or publish has been conducted in ways that are compatible with the three values.

Over 20 practical recommendations, informed by the three values, are made to these duty-bearers. Four key recommendations are summarised in a ‘call for action’, which has been supported by leading research funders and others, including Wellcome, Fiocruz and the African Academy of Sciences.^5^ These institutions have publicly aligned themselves with recommendations to:

- work closely in partnership with emergency responders;
- invest in community engagement mechanisms;
- promote fair collaborations between research institutions in low- and high-income countries; and
- support emergency planning, including helping secure robust health and research systems.

Such commitments are a highly welcome first step to a more holistic approach to research ethics. The next step will be for these commitments to be embedded within the organisational culture of each ‘duty-bearer’. For research funders, for example, this could be through mechanisms such as funding policies, grant application templates and reviewing guidance. Such an approach will provide a sound basis for promoting a fair and respectful relationship between researchers and the communities with whom they work – both in emergencies and in less pressured times.
